# Automated calculation of brain parenchymal fraction as a fast and user-independent method to monitor intracranial CSF volume in hydrocephalus

**DOI:** 10.1186/2045-8118-12-S1-P55

**Published:** 2015-09-18

**Authors:** Johan Virhammar, Marcel Warntjes, Katarina Laurell, Elna-Marie Larsson

**Affiliations:** 1 Department of Neuroscience, Neurology, Uppsala University Hospital, Sweden; 2 Center for Medical Imaging Science and Visualization (CMIV), Linköping University, Linköping, Sweden; 3 SyntheticMR AB, Linköping, Sweden; 4 Department of Pharmacology and Clinical Neuroscience, Umeå University, Sweden; 5 Department of Surgical Sciences, Radiology, Uppsala University, Sweden

## Introduction

Removal of a bolus of CSF by a lumbar puncture (tap test) can be used as a prognostic test in normal pressure hydrocefalus (NPH). The brain parenchymal fraction (BPF) is calculated as (intracranial volume – CSF volume) / intracranial volume. An automatic method to calculate BFP was compared with manual segmentation of lateral ventricular volume as methods to monitor changes in intracranial CSF after a tap test.

## Methods

A lumbar puncture with drainage of 40 mL CSF was performed in 23 patients with idiopathic NPH. Magnetic resonance imaging (MRI) was performed with a 3T scanner with a sequence (QRAPMASTER) allowing quantification of relaxation times. MRI was done at two times before, and at 30 minutes, 4 hours and 24 hours after the tap test. At each investigation time, the volume of the lateral ventricles was manually segmented. BPF was automatically calculated using the post-processing software SyMRI 7.0 (Synthetic MR, Sweden). SyMRI simultaneously measures T1 and T2 relaxation and proton density (PD) values to segment intracranial volume (ICV), gray matter (GM), white matter (WM) and CSF. Summation of the tissues over the complete imaging volume automatically produces GM, WM and CSF volumes in less than 2 minutes.

## Results

At 30 minutes after the lumbar puncture, the volume of the lateral ventricles decreased 5.6±1.9 mL (p<0.0001) using manual segmentation while BPF increased 0.78±0.41% (p<0.001). Differences were significant for both methods also at 4 hours and 24 hours after the tap test. There was a correlation between change in BPF and change in manually segmented ventricular volume with goodness of fit R2=0.45 (p<0.0001).

## Conclusions

BPF is provided rapidly and fully automatically with SyMRI and can be used to monitor changes in intracranial CSF volume. These changes correlate with changes of ventricular volume and may be used for the clinical monitoring of hydrocephalus.
